# Gut microbes associated with functional cure of chronic hepatitis B

**DOI:** 10.1007/s12072-025-10776-9

**Published:** 2025-01-27

**Authors:** Takashi Honda, Masatoshi Ishigami, Yoji Ishizu, Norihiro Imai, Takanori Ito, Kenta Yamamoto, Shinya Yokoyama, Hisanori Muto, Yosuke Inukai, Asuka Kato, Asako Murayama, Sachiyo Yoshio, Tetsuya Ishikawa, Mitsuhiro Fujishiro, Hiroki Kawashima, Takanobu Kato

**Affiliations:** 1 Department of Gastroenterology and Hepatology, Nagoya University Graduate School of Medicine, 65 Tsuruma-cho, Showa-ku, Nagoya, 466-8550 Japan; 2 Department of Virology II, National Institute of Infectious Diseases, Toyama 1-23-1, Shinjuku-ku, Tokyo, 162-8640 Japan; 3 Department of Liver Diseases, The Research Center for Hepatitis and Immunology, National Center for Global Health and Medicine, Kohnodai 1-7-1, Ichikawa, 272-8516 Japan; 4 Department of Gastroenterology, Graduate School of Medicine, The University of Tokyo, Tokyo, Japan

**Keywords:** HBV, Short-chain fatty acids, Butyrate, HBsAg-negative, cccDNA

## Abstract

**Background and aims:**

Hepatitis B virus (HBV) is prevalent worldwide and is difficult to eradicate. Current treatment strategies for chronic hepatitis B ultimately seek to achieve functional cure (FC); however, the factors contributing to FC remain unclear. We aimed to investigate the gut microbiota profiles of patients with chronic hepatitis B who achieved FC.

**Methods:**

Among 105 HBeAg-negative patients with chronic hepatitis B, 70 were enrolled, after excluding patients with cirrhosis or hepatocellular carcinoma and those receiving nucleoside analogs. The gut microbiota of patients who achieved FC was assessed and compared with that of patients with high-titer of HBV DNA (HBV DNA ≥ 3.3 log IU/mL) or low-titer of HBV DNA (HBV DNA < 3.3 log IU/mL). Furthermore, we used cell culture-generated HBV (HBVcc) as a model for HBV infection to evaluate the effects of short-chain fatty acids (SCFAs) produced by the identified bacteria.

**Results:**

There was no difference in the alpha or beta diversity of the gut microbiota between the FC group and the other groups. However, compared with the other groups, the FC group presented a greater relative abundance of bacteria that produce SCFAs, especially butyrate. In vitro studies demonstrated that 1.0 mM butyrate reduces HBsAg production in HBVcc-infected cells. Furthermore, butyrate administration was most effective at the post-HBV infection stage.

**Conclusions:**

Our findings suggest that butyrate-producing bacteria contribute to FC in HBeAg-negative patients with chronic hepatitis B through butyrate-mediated inhibition of HBV production.

**Graphical abstract:**

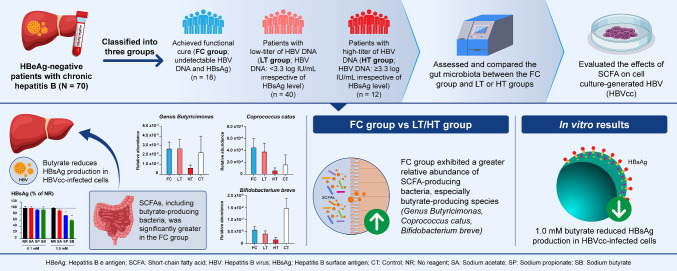

## Key Summary Points

**Table:** 

Bacteria that produce butyrate are abundant in chronic hepatitis B patients who have achieved functional cure, i.e., absence of HBsAg, which is the current goal of chronic hepatitis B treatment.
In vitro, butyrate most efficiently reduced HBV production from HBV-infected cells when administered after infection.

## Introduction

Hepatitis B virus (HBV) affects approximately 290 million people worldwide, and more than one million people die annually from HBV-induced liver failure, cirrhosis, or hepatocellular carcinoma. Therefore, treating HBV is a high priority. Currently, interferon and nucleoside analogs are the available treatments for chronic hepatitis B. However, interferon has various side effects, and orally administered nucleoside analogs must be taken for life (i.e., cannot be discontinued). Therefore, researchers are currently focused on developing therapies that target various points in the HBV life cycle, including entry, replication, encapsidation, and particle release. Since HBV exists in a stable state within the nucleus of human hepatocytes in the form of covalently closed circular DNA (cccDNA), which is a template for pregenomic RNA synthesis, there is no effective treatment for eradicating infected HBV. Rather, the current clinical goal of treatment for chronic hepatitis B is to achieve hepatitis B surface antigen (HBsAg)-negative status, which is designated a functional cure (FC) [1]. Some patients with chronic hepatitis B infection develop cirrhosis or hepatocellular carcinoma, whereas other patients with a good prognosis may develop FC spontaneously without treatment. Accordingly, it is important to elucidate the factors and mechanisms underlying FC during the natural history of chronic hepatitis B to inform treatment interventions for achieving FC.

The human body contains 10–100 times more microbial cells than human cells. These microbes include bacteria, fungi, and viruses, including 100 trillion intestinal bacteria. The human gut microbiota becomes stable at approximately 1 year of age but can subsequently be affected by diet, environmental factors, and exposure to therapies, such as antibiotics or proton pump inhibitors. The development of next-generation sequencing has allowed the detection of bacteria that are difficult to culture. Changes in the intestinal microbiota are involved not only in gastrointestinal diseases but also in diseases of other organs, including the liver. Toll-like receptor ligands, such as lipopolysaccharide, which is a component of the outer cell wall membrane of gram-negative bacteria, have been shown to flow from the portal vein to the liver and promote liver inflammation and fibrosis [2]. The gut microbiota is known to affect host immunity and influence the pathogenesis underlying infectious diseases, including infections caused by enteroviruses, human immunodeficiency viruses, influenza viruses, and HBV [3]. It is also known to be involved in the clearance of HBV in a mouse model of HBV infection [4]. These findings suggest that the gastrointestinal tract and liver are closely related and constitute the gut‒liver axis [5], [6]. Therefore, we conducted an exploratory study focusing on gut microbiota profiles that were specific for patients with chronic hepatitis B who achieved FC and investigated whether the gut microbiota is involved in naturally achieved FC among patients with chronic hepatitis B.

## Materials and methods

### Patients

A total of 105 patients with hepatitis B who visited Nagoya University Hospital between April 2019 and March 2021 provided consent to participate in this study. After patients with cirrhosis or hepatocellular carcinoma and those who received nucleoside analog treatment were excluded, 70 HBeAg-negative patients were included. Cirrhosis was diagnosed by ultrasound examination and/or dynamic contrast-enhanced computerized tomography. The enrolled patients were divided into three groups: 18 patients who achieved FC (FC group; undetectable HBV DNA and HBsAg), 40 patients with low-titer of HBV DNA (LT group; HBV DNA: < 3.3 log IU/mL irrespective of HBsAg level), and 12 patients with high-titer of HBV DNA (HT group; HBV DNA ≥ 3.3 log IU/mL irrespective of HBsAg level). Eight volunteers (median age 46 years) were enrolled as healthy controls. This study was approved by the Ethics Committee of Nagoya University Hospital (approval numbers: 2017–0038) and was conducted in accordance with the Declaration of Helsinki (1975).

### Virological markers

The HBV DNA titer was measured via the COBAS AmpliPrep/COBAS TaqMan HBV Test v2.0 (Roche Diagnostics, Tokyo, Japan). The dynamic range of this assay was 2.1–9.0 log copies/mL. The titers of HBsAg (Architect HBsAg QT; Abbott Japan, Tokyo, Japan), HBeAg (Architect HBeAg; Abbott Japan), hepatitis B core-related antigen (HBcrAg; Fujirebio, Tokyo, Japan), and anti-HBe (Architect HBeAb; Abbott Japan) were examined via chemiluminescence immunoassays. The lower detection limits of HBsAg and HBcrAg are 0.05 IU/mL and 3.0 log U/mL, respectively.

### Analysis of the gut microbiota

Stool samples were collected using special containers for gut microbiota analysis (TechnoSuruga Laboratory, Shizuoka, Japan). Collection was stopped if the patient had taken antibiotics. The V3–4 region of the 16S rRNA gene was amplified in DNA extracted from feces and analyzed using an Illumina MiSeq. For the annotation of 16S rRNA gene sequences, we used the Greengenes database, which is renowned for its comprehensiveness and regular updates suitable for microbial diversity studies. Annotations were performed with QIIME2 (version 2019.11 https://docs.qiime2.org/2020.11/) [7], applying the DADA2 pipeline for sequence quality control, which includes stringent filtering, denoising, and chimera removal steps. Linear discriminant analysis effect size (LEfSe) [7] was also used to assess differences in the gut microbiota, as previously described [8]. The relative abundances of bacterial groups that showed significant differences in the intestinal microbiota between the FC and the LT or HT groups were determined. To confirm whether these between-group differences in relative abundance were associated with changes in pathological conditions, we determined the relative abundance of the gut microbiota in the feces of the healthy control (CT) group.

### Cell culture and reagents

HepG2 cells transduced with sodium taurocholate cotransporting polypeptide (HepG2–NTCP cells) were used as previously described [9], [10]. Human primary hepatocytes (PXB cells) were obtained from Phoenix Bio (Hiroshima, Japan) and cultured according to the manufacturer’s instructions [11]. PXB cells were isolated from primary human hepatocyte-transplanted urokinase-type plasminogen activator/severe combined immunodeficiency mice (PXB mice, PhoenixBio). Short-chain fatty acids (SCFAs), namely, sodium acetate (SA), sodium propionate (SP), and sodium butyrate (SB), were purchased from FUJIFILM Wako Pure Chemical (Osaka, Japan).

### Cell culture-generated HBV

Cell culture-generated HBV (HBVcc) was produced via the transfection of a plasmid encoding a replication-competent HBV clone with a 1.38-fold genome length (genotype C, accession number: AB246344) into HepG2/NTCP cells using Lipofectamine 3000 Reagent (Thermo Fisher Scientific, Waltham, MA) as previously described [9], [11]. This clone possesses an amino acid substitution, G1896A, which prevents HBeAg production. The generated HBVcc was concentrated and purified via an iodixanol density gradient as previously described [9], and the peak fraction of infectivity in the gradient was used as an inoculum. The HBsAg levels in the culture medium of HBVcc-infected cells were measured via a chemiluminescent enzyme immunoassay (Lumipulse, Fujirebio, Tokyo, Japan) [12].

### HBVcc infection assay

PXB cells were pretreated with SCFAs 1 day before infection. Before infection, purified HBVcc was mixed with SCFAs and incubated at 37 °C for 1 h. SCFA-treated HBVcc was inoculated into PXB cells at 100 GEq per cell in the presence of 4% PEG8000 for 16 h. The infected cells were cultured with SCFAs, and HBsAg levels were monitored. After 11 days of culture, infected cells were visualized by staining with a rabbit polyclonal anti-HBc antibody (AUSTRAL Biologicals, San Ramon, CA, USA) and Alexa Fluor 555-conjugated anti-rabbit IgG (Thermo Fisher Scientific). Nuclei were stained with 4',6-diamidino-2-phenylindole (DAPI). The experiments were performed in triplicate and repeated at least two times.

### Statistical analysis

Continuous variables are expressed as medians (interquartile ranges) and were analyzed via the Kruskal‒Wallis test. Categorical variables were analyzed via the chi-square test or Fisher’s exact test. Statistical significance was set at *p* < 0.05. All statistical analyses were performed with SPSS software (version 26.0; SPSS Japan, Tokyo, Japan).

Microbiome comparisons and diversity were visualized and statistically analyzed using the online microbiome data analysis platform Microbiome Analyst (https://www.microbiomeanalyst.ca/MicrobiomeAnalyst) [13]. The settings were as follows: low-count filter (minimum count: 0, percentage to remove: 0%) and data rarefying (data rarefying: rarefy to the minimum library size). Alpha diversity was calculated on the basis of the Shannon index via the Kruskal‒Wallis test. Beta diversity was estimated via the unweighted UniFrac distance and was visualized using the principal coordinate analysis (PCoA). The significance of the PCoA results was analyzed using permutational multivariate analysis of variance, which uses distance metrics based on the Bray‒Curtis index to confirm the strength and statistical significance of the sample groupings. Between-group differential taxonomy was analyzed via linear discriminant analysis effect size (LEfSe:http://huttenhower.sph.harvard.edu/galaxy/) [7], with a linear discriminant analysis (LDA) score > 2 and a *p* value of < 0.05 considered to indicate significantly different populations.

## Results

### Patient backgrounds

HBsAg and HBV DNA were undetectable in the FC group. The median HBsAg and HBV DNA levels were lower in patients in the LT group than in those in the HT group. The median age was significantly greater in the FC group than in the HT group (*p* < 0.05). The median ALT and Alb levels in the FC group were significantly lower than those in the HT group (ALT, *p* < 0.001; Alb, *p* < 0.05). The median HBcrAg level in the FC and LT groups was undetectable. There were no significant among-group differences in the presence or absence of diabetes mellitus, probiotics, or proton pump inhibitor administration, all of which may affect intestinal bacterial communities (Table 1).

**Table 1:** Baseline clinical characteristics of the patients

	FC (*n* = 18)	LT (*n* = 40)	HT (*n* = 12)
Sex [male/female]	7/11	22/18	8/4
Age (year)	65.0 * (53.0–68.0)	53.0 (44.0—68.0)	51.0 (42.8—55.8)
ALT (IU/L)	15.0 ** (12.0–21.0)	21.0 (14.3–25.8)	30.0 (20.8–49.3)
γGTP (IU/L)	19.5 (13.0–23.0)	19.0 (14.0–29.0)	26.0 (18.3–36.3)
T-Bil (mg/dL)	0.9 (0.6–1.2)	0.8 (0.7–1.0)	0.9 (0.7–1.3)
Albumin (g/dL)	4.2* (4.1–4.4)	4.3 (4.2–4.4)	4.6 (4.2–4.8)
Platelet count (× 10^4^/μL)	20.4 (17.1–23.5)	21.3 (19.2–23.5)	24.1 (18.7–26.7)
HBsAg (IU/mL)	ND	481.7 (18.1–2846.5)	2599.5 (639.0–6379.7)
HBV DNA (log IU/mL)	ND	2.4 (1.7–2.9)	4.4 (3.7–5.0)
HBcrAg (log U/mL)	< 3.0 (< 3.0–< 3.0)	< 3.0 (< 3.0–< 3.0)	3.3 (< 3.0–4.2)
Diabetes mellitus [presence/absence]	0/18	0/40	2/10
Probiotics [presence/absence]	0/18	0/40	0/40
Proton pump inhibitor [presence/absence]	0/18	2/38	1/11

Continuous variables are expressed as medians (interquartile ranges) and were analyzed via the Kruskal‒Wallis test

*ALT* alanine aminotransferase, *γGTP* gamma-glutamyl transpeptidase, *T-Bil* total bilirubin, *HBsAg* hepatitis B surface antigen, *HBcrAg* hepatitis B core-related antigen, *ND* not detected. *p < 0.05, **p < 0.001 compared with the HT group

### Gut microbial diversity

**Fig. 1:**
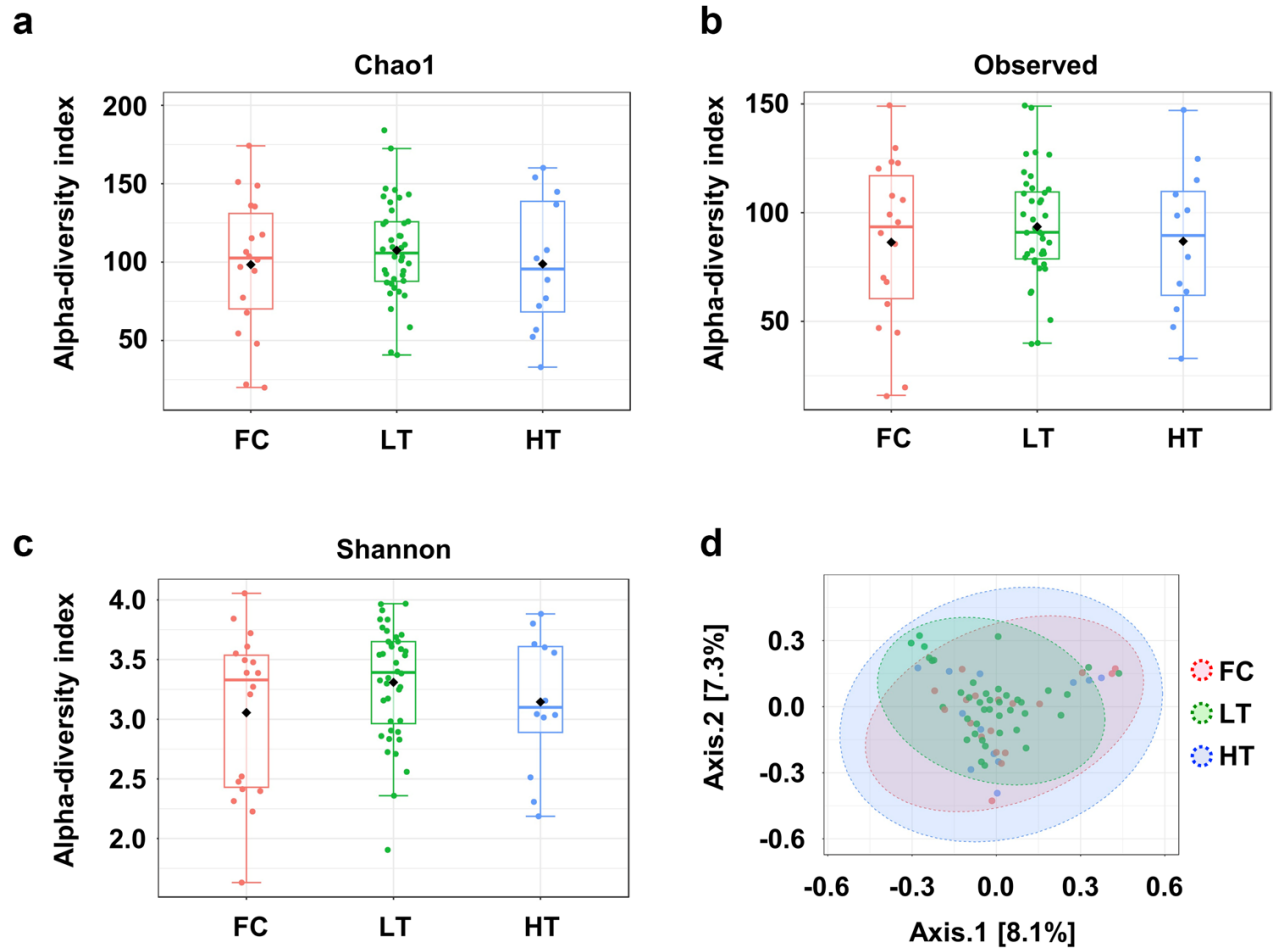
Diversity of the gut microbiota in functional cure. The alpha diversities of the gut microbiota in patients in the FC, LT, and HT groups were analyzed using the Chao1 (**a**), observed (**b**), and Shannon index methods (*p* > 0.05). **d** Beta diversities of the gut microbiota in patients in the FC, LT, and HT groups were analyzed via PCoA. The PCoA plot illustrates the relationships and distances between microbial communities in different groups (*p* > 0.05)

To assess the diversity of the gut microbiota in relation to the achievement of FC, we compared several microbial diversity parameters across groups. We found no significant differences in alpha diversity, as measured by the Chao1, Observed, and Shannon indices, between the groups (Fig. 1A, B, C). For beta diversity analysis, we employed PCoA. The PCoA plot illustrates the relationships and distances between microbial communities in different groups. No significant difference was observed among these groups (Fig. 1D).

### Differences in the gut microbiota

**Fig. 2:**
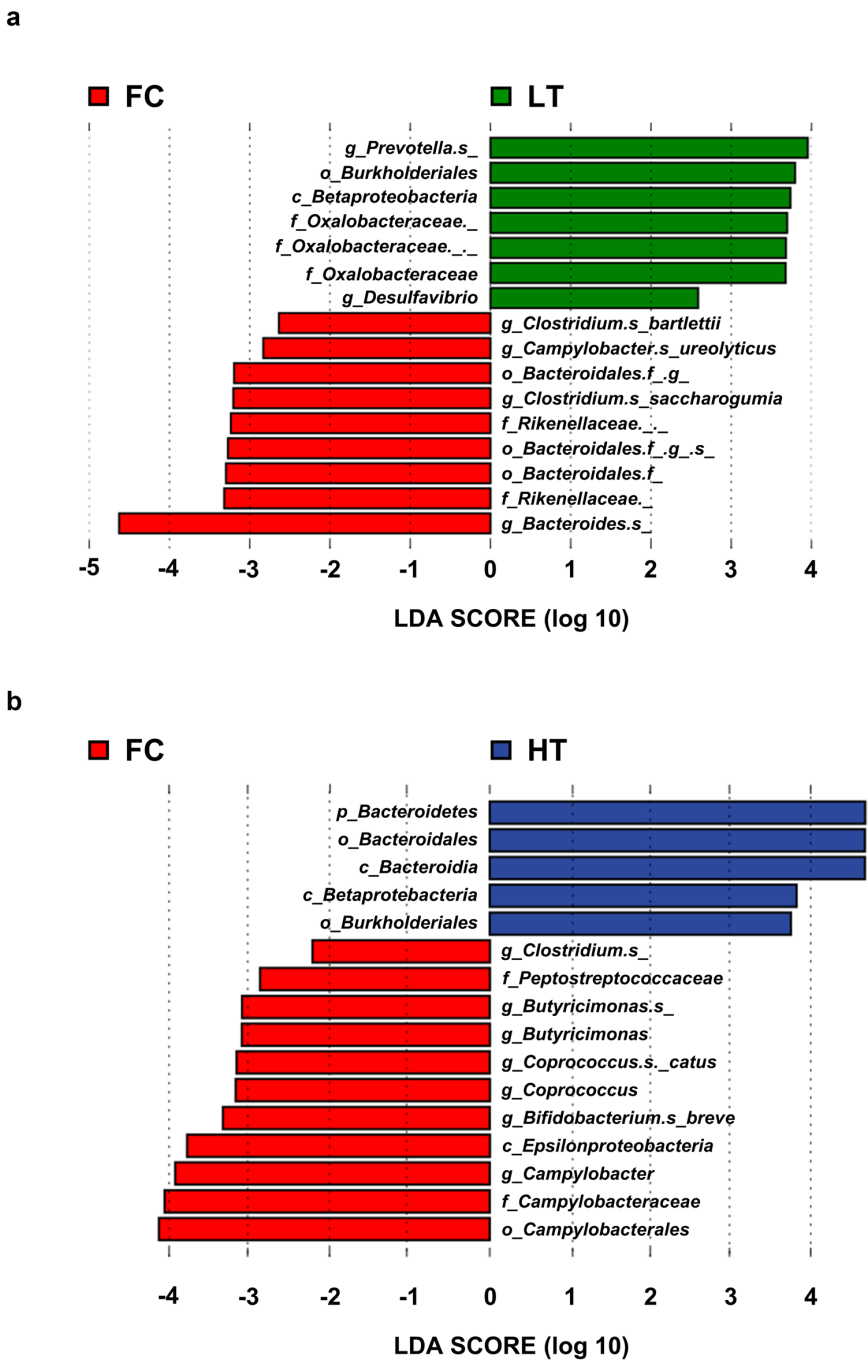
Analysis of microbial abundance in FC and hepatitis patients. The microbiota in each group were analyzed by LEfSe. Histograms of the linear discriminant analysis (LDA) scores between FC and LT (**a**) or between FC and HT (**b**) are shown

Next, the relative abundance of bacteria in the gut microbiota was analyzed via LEfSe and compared between the FC and both the LT and HT groups. In the analysis between the FC and LT groups, seven and nine bacteria were significantly abundant in patients with LT and FC, respectively (Fig. 2A). In the analysis between the FC and HT groups, five and eleven bacteria were significantly abundant in patients with HT and FC, respectively (Fig. 2B). Among the identified bacteria, we further focused on bacteria whose abundance gradually increased with the improvement of the disease condition, specifically, *Clostridium bartlettii*, *Genus Butyricimonas., Coprococcus catus*, *Bifidobacterium breve,* and *Genus Campylobacter*.

### Gut microbiota associated with FC

**Fig. 3:**
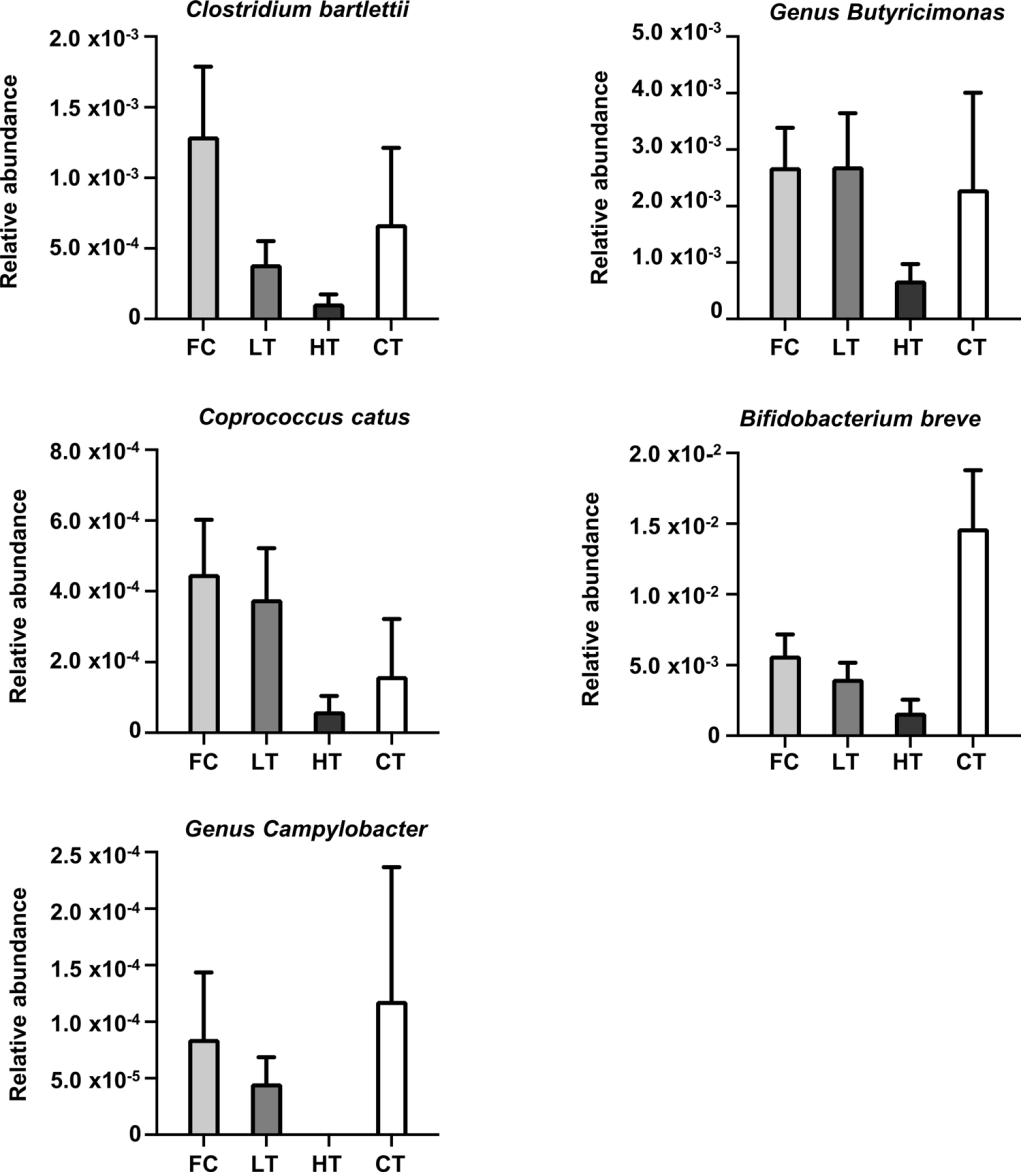
Gut microbiota associated with FC. The relative abundances of *Clostridium bartlettii*, *Genus Butyricimonas.*, *Coprococcus catus*, *Bifidobacterium breve*, and *Genus Campylobacter* in the FC, LT, HT, and CT groups are indicated

The abundances of the five identified bacteria in patients in the FC, LT, and HT groups were compared with those in the CT group. The abundances of these bacteria were greater in the FC group than in the HT group and were also high in the CT group (Fig. 3). Among the five bacteria identified, three, *Genus Butyricimonas., Coprococcus catus, and Bifidobacterium breve*, are known to produce SCFAs, especially butyrate. Therefore, we reasoned that butyrate production and FC might be associated and evaluated the effects of SCFAs, including butyrate, on the HBV life cycle.

### Effects of SCFAs on the HBV life cycle

**Fig. 4:**
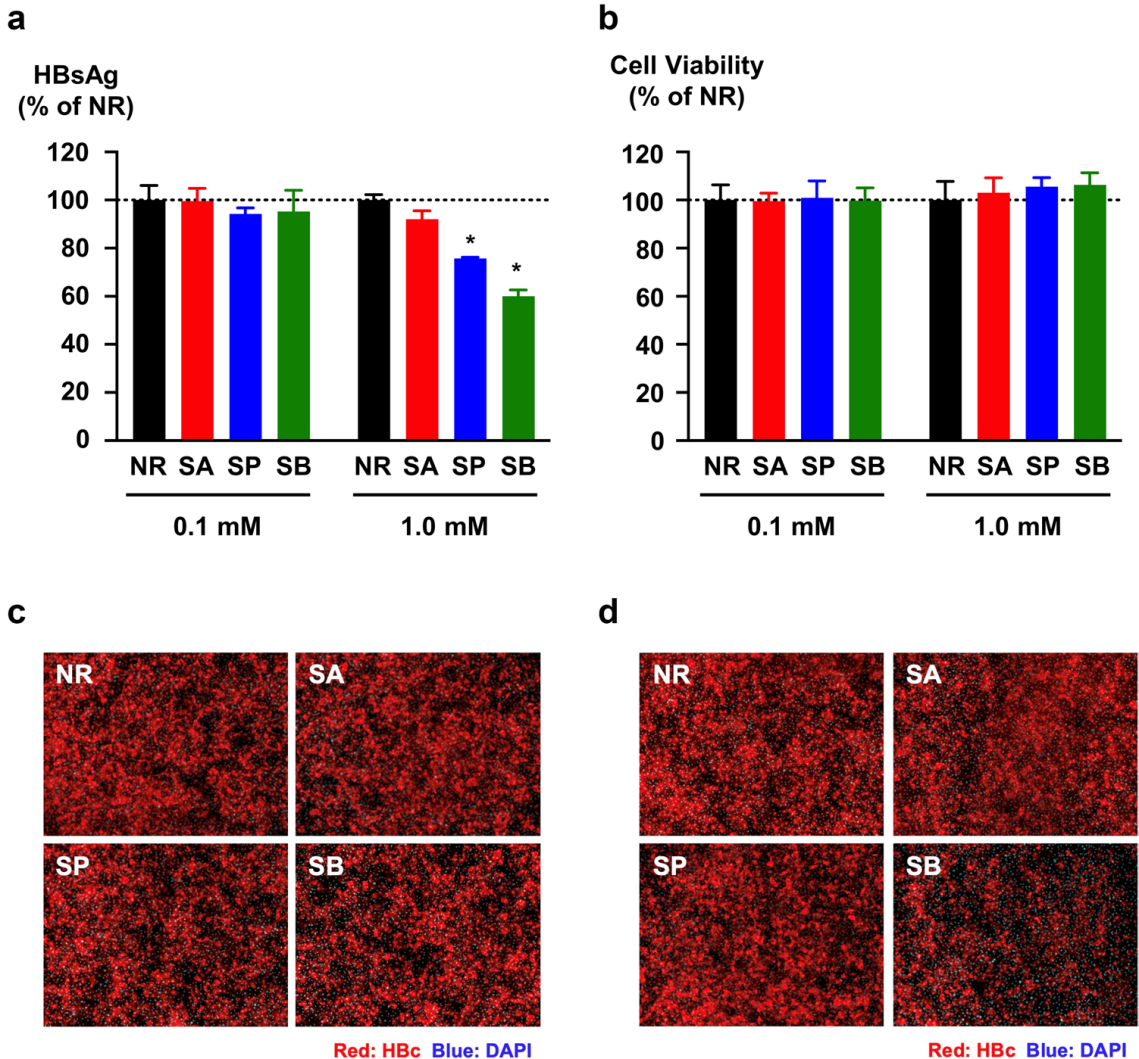
Effects of SCFAs on HBVcc infection. **a** Effects of SCFAs on HBsAg production were evaluated. PXB cells were incubated with the indicated SCFAs (0.1 or 1.0 mM) for 24 h prior to infection. The HBVcc was treated with SCFAs (0.1 or 1.0 mM) at 37 °C for 1 h before infection and infected with PXB cells at 100 GEq/cell. After 16 h of infection, the infected cells were cultured with SCFAs (0.1 or 1.0 mM) for 11 days. The HBsAg levels in the culture media were measured at the end of the observation period and are presented as a percentage of the level in the NR group. *p < 0.01 compared with NR. **b** Viability of SCFA-treated cells was evaluated via the WST-8 assay. The cell viability is presented as a percentage of that in the NR group. **c**, **d** Effects of SCFAs on HBV infection. PXB cells were pretreated with SCFAs at 0.1 mM (**c**) and 1.0 mM (**d**), and HBVcc treated with SCFAs at 0.1 mM (**c**) and 1.0 mM (**d**) were infected. The infected cells were cultured with SCFAs at 0.1 mM (**c**) and 1.0 mM (**d**) for 11 days. HBc-positive cells were detected via immunostaining with an anti-HBc antibody

To assess the effects of SCFAs on the HBV life cycle, we used an HBV infection system in a cell culture. PXB cells were pretreated with SCFAs, including SA, SP, and SB. Then, HBVcc was treated with SCFAs for 1 h at 37 °C and infected with SCFA-treated PXB cells. In addition, infected cells were cultured with SCFAs for 11 days. The HBsAg levels in the culture medium of infected cells were measured and compared with those in cells not treated with SCFAs (no reagent; NR). The HBsAg levels did not differ between the NR group and the group treated with 0.1 mM SCFAs. However, 1.0 mM SB treatment reduced the HBsAg level to 60.0 ± 2.6% of the control level (NR), and SP treatment reduced the HBsAg level to 75.8 ± 0.5% of the control level. However, no detectable reduction was caused by the SA treatment (Fig. 4A). Immunostaining revealed that the number of HBc-positive cells was reduced by 1.0 mM SB treatment; however, minimal reduction in the number of HBc-positive cells was detectable upon 0.1 mM SCFA treatment (Fig. 4B, C).

### Affecting point of SCFAs on the HBV life cycle

**Fig. 5:**
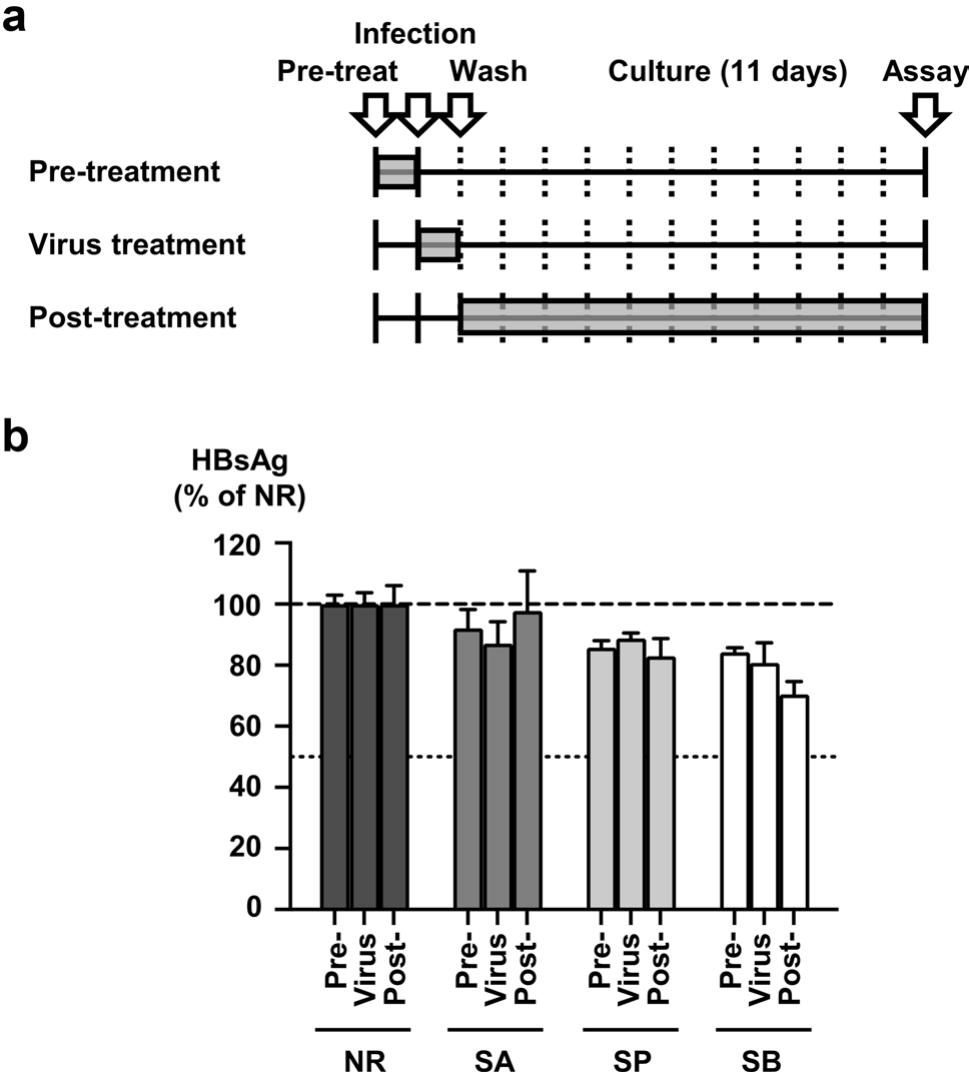
Affecting point of SCFAs on the HBV life cycle. **a** Affecting point of SCFAs on the HBV life cycle was evaluated. Three SCFA treatment periods were studied: PXB cells were incubated with SCFAs (1.0 mM) for 24 h prior to infection (pre-treatment). HBVcc was treated with SCFAs (1.0 mM) at 37 °C for 1 h before infection and included SCFAs during infection (virus treatment). At 16 h after infection, the infected cells were cultured with 1.0 mM SCFAs for 11 days (post-treatment). **b** HBsAg levels in the culture medium of the pre-treatment, virus treatment, and post-treatment groups were measured on day 12 postinfection. The HBsAg levels are presented as a percentage of those in the NR group

To clarify the timepoint at which SCFAs affect the HBV life cycle, treatments were administered at three different periods: prior to infection (pre-treatment), during infection (viral treatment), and after infection (post-treatment). The SCFAs were administered in each of these phases as follows: pre-treatment: SCFA treatment of PXB cells for 24 h prior to infection; virus treatment: SCFA treatment of HBVcc at 37 °C for 1 h before infection and during infection; and post-treatment: SCFA administration in culture for 11 days after infection (Fig. 5A). A substantial reduction in HBsAg levels was detected after SB treatment and again after SP treatment. Specifically, compared with NR, SB treatment during the post-treatment phase reduced the HBsAg level to 70.4 ± 4.2% of the control level (NR). SB treatment during the pre-treatment phase reduced the HBsAg level to 84.3 ± 1.4% of the control level. SB treatment during the virus treatment step was reduced to 80.8 ± 6.6% of the control level. Thus, among the three treatment periods tested, SB administration in the post-treatment phase was the most effective. Likewise, among the three treatment periods of SP administration, SP treatment during the post-treatment phase was the most effective approach and reduced the HBsAg level to 82.9 ± 5.8% of the control level.

## Discussion

SCFAs produced by intestinal bacteria, including acetate, propionate, and butyrate, have various health benefits, such as keeping the intestinal tract mildly acidic, inhibiting the growth of harmful bacteria, preventing obesity and intestinal inflammation, and regulating the immune system. In this study, we hypothesized that changes in the gut microbiota might be associated with the achievement of FC of hepatitis B. Thus, we investigated the differences in the gut microbiota between patients who achieved FC and those with active or stable chronic hepatitis B. The results revealed that certain bacteria were more abundant in FC patients than in LT or HT patients.

The gut microbiota is closely related to pathophysiology and disease progression in chronic HBV infection, specifically, a reduction in SCFA-producing bacteria has been observed in HBV-infected patients [14]. In this study, we identified five bacterial species as possibly associated with the achievement of FC. Among them, *Genus Butyricimonas., Coprococcus catus, and Bifidobacterium breve* produce SCFAs. *Genus Butyricimonas* are known to produce butyrate [15], [16]; furthermore, the abundance of *Genus Butyricimonas.* is increased by the administration of probiotics and contributes to an increase in the fecal concentration of butyrate [17]. *Coprococcus catus* belongs to the Lachnospiraceae family and has also been reported to produce butyrate [18], [19]. *Bifidobacterium breve* is a type of bifidobacterium that produces lactic acid and acetic acid, which may contribute to increased butyrate levels [20], [21]. The relative abundance of Bifidobacteria is known to be age-dependent; its abundance is high up to the age of 1 year and decreases with age. In our study, the median age was highest in the FC group, followed by the LT group. The median age of the CT group was the lowest 46.0 years. Therefore, the abundance of *Bifidobacterium breve* in the FC group was expected to be lower than that in the other groups. However, a higher abundance of *Bifidobacterium breve* was observed in the FC group than in the LT and HT groups. These findings imply that it is likely a distinctive bacterium associated with the achievement of FC. Notably, in an in vivo experiment using a colitis mouse model, oral administration of *Bifidobacterium breve* was found to increase SCFA levels in the colon and improve colitis [22]. With respect to the association between SCFAs and hepatitis B, HBeAg-positive chronic hepatitis B patients with a low abundance of butyrate-producing bacteria have been reported to achieve lower HBeAg levels after fecal transplantation from healthy donors with high levels of butyrate-producing bacteria [23]. These observations suggest that butyrate-producing bacteria contribute to an enhanced immune response or the reduction of HBV levels and are consistent with our findings, suggesting a relationship between the abundance of butyrate-producing bacteria and the improvement of hepatitis in chronic hepatitis B patients. Therefore, it is conceivable that SCFAs produced by the identified bacteria, and especially butyrate, may help to improve hepatitis and promote FC in patients with chronic hepatitis B. Future studies will be needed for the detailed mechanisms of anti-HBV effects associated with SCFAs.

To clarify the mechanism by which SCFAs ameliorate hepatitis B, we focused on the anti-HBV effect of SCFAs, evaluating this effect in an in vitro HBV infection system. HBVcc-infected cells were treated with a variety of SCFAs, and butyrate exhibited the most potent anti-HBV effects. Specifically, butyrate administration at a concentration of 1.0 mM reduced HBsAg production to approximately 60% of that in the no-reagent control; however, such effects were not observed when butyrate was administered at a concentration of 0.1 mM. Propionate was also found to exert an anti-HBV effect, albeit a weaker effect than butyrate. These SCFAs were most effective when administered after HBV infection. These data indicate that the anti-HBV effect of SCFAs is mediated by a reduction in HBV production following HBV infection rather than the inhibition of HBV infection. Such a reduction in HBV production by SCFAs may be one of the mechanisms by which FC associated with SCFA-producing bacteria is achieved. One possible mechanism for the butyrate-associated reduction in HBV production could be through inhibition of the expression of SIRT-1, a class III histone deacetylase. The reduction of the SIRT-1 expression by butyrate has been reported to inhibit HBV replication [24]. Moreover, increased butyrate levels have been shown to suppress intestinal inflammation by promoting the differentiation of immature T cells into regulatory T (Treg) cells [25]. The inhibition of histone deacetylases (HDACs) by butyrate has been suggested to be a mechanism for promoting Treg differentiation via regulating gene expression epigenetically [26], [27]. Although the relationship between the promotion of Treg differentiation and FC remains unclear, the inhibition of HDACs may be one of the mechanisms underlying the butyrate-mediated reduction in HBV production in HBV-infected cells, because reduction of HBV replication by inhibition of HDAC has been reported in duck HBV model [28]. The SCFA administration in the post-treatment phase was the most effective to reduce HBsAg production in HBV infected cells. These data imply that the SCFA suppresses the expression of HBV-related proteins in infected cells and, as a result, reduces the efficiency of cccDNA recycling, thereby promoting the achievement of FC.

The concentrations of SCFAs exhibited anti-HBV effects in the in vitro study seemed to be relatively high. In human samples obtained within 4 h of death, SCFA concentrations were reported to be approximately 100 mM, 400 μM, and 150 μM in the intestinal tract, portal vein, and liver, respectively [29]. Another study reported that SCFA concentrations ranged from 50 to 150 mM in the colon and that acetic acid, propionic acid, and butyric acid were present in a 3:1:1 ratio, but the concentrations in the portal vein or liver may be lower [18]. However, even relatively low concentrations of SCFAs produced by bacteria may be sufficient to reduce HBV if the SCFAs produced in the intestinal tract act on HBV in the liver through the portal vein for an extended period. This idea is consistent with the fact that a long period is needed to achieve FC clinically.

In summary, we observed no difference in the α and β diversity among the LT, HT, and FC groups. However, the relative abundance of SCFA-producing bacteria, especially butyrate-producing bacteria, was significantly greater in the FC group. Moreover, the relative abundance of such bacteria gradually increased with clinical improvement. These data suggest that the abundance of butyrate produced by the gut microbiota may be associated with improvements in the clinical status of chronic hepatitis B and the achievement of FC. In addition, as one of the mechanisms for achieving FC associated with butyrate-producing bacteria, the direct anti-HBV effects of butyrate were clarified.

## Data Availability

Data will be made available upon request.

## Abbreviations

HBV: Hepatitis B virus
FC: Functional cure
HBVcc: Cell culture-generated HBV
SCFAs: Short-chain fatty acids
HBsAg: Hepatitis B surface antigen
HBeAg: Hepatitis B e antigen
LT: Low-titer of HBV DNA
HT: High-titer of HBV DNA
HBcrAg: Hepatitis B core-related antigen
SA: Sodium acetate
SP: Sodium propionate
SB: Sodium butyrate
DAPI: 4',6-Diamidino-2-phenylindole
